# Exenatide once weekly treatment maintained improvements in glycemic control and weight loss over 2 years

**DOI:** 10.1186/1472-6823-11-9

**Published:** 2011-04-29

**Authors:** Kristin Taylor, Kate Gurney, Jenny Han, Richard Pencek, Brandon Walsh, Michael Trautmann

**Affiliations:** 1 Amylin Pharmaceuticals, Inc., 9360 Towne Centre Drive, San Diego, CA 92121, USA; 2 Eli Lilly and Company, Lilly Corporate Center: DC 6038, Indianapolis, IN 46285, USA

## Abstract

**Background:**

The once-weekly (QW) formulation of the glucagon-like peptide-1 receptor agonist exenatide has been demonstrated to improve A1C, fasting plasma glucose (FPG), body weight, serum lipid profiles, and blood pressure in patients with type 2 diabetes through 52 weeks of treatment. In this report, we describe the 2-year results of the open-label, open-ended extension to the DURATION-1 trial of exenatide QW for type 2 diabetes.

**Methods:**

A 2-stage protocol was used: patients received either exenatide QW (2 mg) or exenatide twice daily for 30 weeks (5 μg for the first 4 weeks and 10 μg thereafter), followed by 1.5 years of treatment with exenatide QW (2 mg), for a total of 2 years (104 weeks) of exenatide treatment. Of the 295 (intent-to-treat [ITT]) patients who entered the trial, 73% (n = 216) completed 2 years of treatment (completer population). Baseline characteristics (mean ± SE) for these patients were: A1C, 8.2 ± 0.1%; FPG, 168.4 ± 43.0 mg/dL; body weight, 101.1 ± 18.7 kg; and diabetes duration, 7 ± 5 years.

**Results:**

In the completer population, significant improvements (LS mean ± SE [95% CI]) were maintained after 2 years of treatment in A1C (-1.71 ± 0.08% [-1.86 to -1.55%]), FPG (-40.1 ± 2.9 mg/dL [-45.7 to -34.5 mg/dL]), and body weight (-2.61 ± 0.52 kg [-3.64 to -1.58 kg]) compared with baseline. The percentages of patients who achieved an A1C of <7.0% and ≤6.5% at 2 years were 60% and 39%, respectively. A significant reduction in systolic blood pressure (SBP; -3.0 ± 1.0 mmHg [-4.9 to -1.1 mmHg]) was maintained through 2 years of treatment. Serum lipid profiles were also significantly improved, including triglycerides (geometric LS mean change from baseline, -15 ± 2.7% [-21% to -10%]), total cholesterol (-8.6 ± 2.8 mg/dL [-14.0 to -3.1 mg/dL]), and low-density lipoproteins (-4.5 ± 2.2 mg/dL [-8.9 to -0.01 mg/dL]). Changes in A1C, body weight, FPG, SBP, and lipids in the ITT population were similar to those seen in the completer population. Nausea (predominantly mild in intensity) was the most common adverse event, although the frequency and intensity of nausea decreased over time. No severe hypoglycemia was observed.

**Conclusions:**

Exenatide QW was well tolerated during the 2-year treatment period. This study demonstrated sustained glucose control and weight loss throughout 2 years of treatment with exenatide QW.

**Trial Registration:**

ClinicalTrials.gov NCT00308139

## Background

Type 2 diabetes is characterized by progressive insulin resistance and pancreatic beta-cell dysfunction resulting in key defects in insulin secretion and function [1]. Ultimately, this leads to difficulties in controlling hyperglycemia, which has been associated with microvascular and macrovascular complications [2,3]. Because lowering of A1C has been shown to reduce the risk of long-term microvascular - and possibly macrovascular - complications [3-7], a target A1C level of <7% has been recommended by the American Diabetes Association (ADA) [8] for patients with diabetes; the American Association of Clinical Endocrinologists and the American College of Endocrinology (AACE/ACE) [9] have recommended a more stringent A1C target of ≤6.5%. Thus, the overall goal for the treatment of type 2 diabetes is to reduce A1C to as near normal levels as possible, with considerations for the risk of hypoglycemia and the risk of comorbidites that are common in type 2 diabetes, including obesity, dyslipidemia, and hypertension.

Treatment of type 2 diabetes often begins with lifestyle modifications, but as the disease progresses, most patients require pharmacotherapy. However, many patients with type 2 diabetes do not maintain the recommended glycemic targets over time with traditional therapies and must transition to combination therapy and/or increasing doses of insulin as their glycemic control deteriorates [5,6,10,11]. Furthermore, the use of many antihyperglycemic agents can be accompanied by increased hypoglycemia, weight gain, and a persistence of elevated postprandial glucose excursions [3]. To address the need for more comprehensive treatment of type 2 diabetes, recent algorithms developed by the AACE and ADA/European Association for the Study of Diabetes have recommended the use of glucagon-like peptide-1 (GLP-1) receptor agonists (monotherapy or combination therapy) on the basis of their effective glycemic control, weight loss effects, low rates of hypoglycemia, and overall safety profiles, and particularly for use in patients for whom hypoglycemia is especially undesirable or promotion of weight loss is a major consideration [2,9,11].

Exenatide is a potent GLP-1 receptor agonist with multiple glucoregulatory effects for the treatment of type 2 diabetes [12-18]. Treatment with exenatide twice daily (BID) enhances glucose-dependent insulin secretion, reduces inappropriate elevations in glucagon secretion, slows gastric emptying, and reduces body weight in a substantial proportion of treated patients [13-19]. Furthermore, the clinical effects of exenatide are sustained over time, as shown in long-term studies of exenatide BID [19,20].

The sustained-release formulation of exenatide, exenatide once-weekly (QW), allows for continuous drug release over time, resulting in sustained therapeutic levels of exenatide - and thus, 24-hour glycemic control - with a single weekly injection [21,22]. Providing continuous GLP-1 receptor agonism has emerged as an increasingly important opportunity in treating type 2 diabetes, as evidence indicates that controlling both fasting and postprandial hyperglycemia is essential to providing comprehensive glycemic control [23,24].

The DURATION-1 trial was designed to compare the safety and efficacy of exenatide BID with exenatide QW in patients with type 2 diabetes. This 2-stage protocol consisted of a randomized, open-label comparison of exenatide QW and exenatide BID for 30 weeks, followed by an open-ended assessment period in which all patients received exenatide QW. The results from the 30- and 52-week assessment periods have previously been reported [25,26]. The objective of this report is to describe the safety and efficacy of 2 years of exenatide QW treatment in patients with type 2 diabetes.

## Methods

### Randomization and interventions

Patients were randomized to one of two open-label treatment groups: 1) weekly subcutaneous (SC) injections of 2 mg exenatide QW or 2) SC injections of exenatide 5 μg BID for the first 4 weeks followed by a dose increase to 10 μg BID for the remainder of the 30-week assessment period. The inclusion and exclusion criteria have previously been reported [25]. At 30 weeks, participants entered an open-ended treatment period in which all patients received exenatide QW. Patients who were originally randomized to exenatide BID were switched to exenatide QW, and those originally randomized to exenatide QW continued their treatment regimens. Patients who were switched to exenatide QW at week 30 and who were concomitantly using a sulfonylurea (SFU) were required to reduce the SFU dose to the minimum recommended dose until week 40. Subsequently, the SFU dose was titrated up based on daily glucose measurements to a target fasting plasma glucose (FPG) of ≤110 mg/dL.

A common clinical protocol was approved for each study site by the appropriate Institutional Review Board. Patients provided written informed consent prior to participation. The study was conducted in accordance with the principles described in the Declaration of Helsinki, including all amendments through the South Africa revision of 1996 [27].

### Outcomes

The objectives of this study were to examine the effects of exenatide QW on glycemic control, body weight, fasting lipids, and blood pressure after 2 years of treatment. Efficacy, safety, and tolerability were assessed in all patients, including intent-to-treat (ITT) and completer populations. Plasma analytes and A1C were quantitated by Quintiles Laboratories (Smyrna, GA) using standard methods [25].

### Statistical analysis

All randomized patients who received at least one injection of exenatide in the 30-week phase of this study were defined as the ITT population. The 2-year (104-week) completer population consisted of patients from the ITT population who completed the study procedures through week 100 in compliance with the protocol and who received at least 96 weeks of treatment. At week 52, there were no significant differences in change from baseline A1C or the percentage of patients who achieved target A1C between the group initially randomized to exenatide QW and the group initially randomized to exenatide BID (who switched to exenatide QW at week 30) [26]. Thus, in this 2-year assessment, the completer population consisted of all patients regardless of initial randomization. Descriptive statistics on demographics and analyses of primary glycemic endpoints, body weight, blood pressure, and fasting lipid concentrations are provided for both the completer and ITT populations. For ITT analyses, the last observation carried forward (LOCF) method was applied to include data collected from the early withdrawals. The analyses of A1C were based on a general linear model (ANOVA) including original treatment assignment, baseline A1C strata, and concomitant SFU use at screening as factors. Baseline FPG, body weight, and blood pressure were added in the model (ANCOVA) for each respective parameter. Efficacy endpoint results are expressed as least square (LS) means. Statistical analysis was performed using SAS (8.2; SAS Institute, Inc., North Carolina).

Treatment-emergent adverse events (TEAEs) were defined as events that occurred during or after the first injection of randomized treatment. Hypoglycemic episodes were classified as major (i.e., severe) if 1) the event resulted in loss of consciousness, seizure, coma, or other change in mental status consistent with neuroglycopenia (as judged by the investigator or physician), in which symptoms resolved after administration of intramuscular glucagon or intravenous glucose, or 2) the event required third-party assistance because of severe impairment in consciousness or behavior and was accompanied by a blood glucose concentration of <54 mg/dL (3.0 mmol/L). Minor hypoglycemia was defined as a report of symptoms consistent with hypoglycemia and a glucose value of <54 mg/dL prior to treatment of the episode. Safety analyses are provided for the ITT population.

## Results

### Patient disposition and baseline characteristics

**Figure 1:**
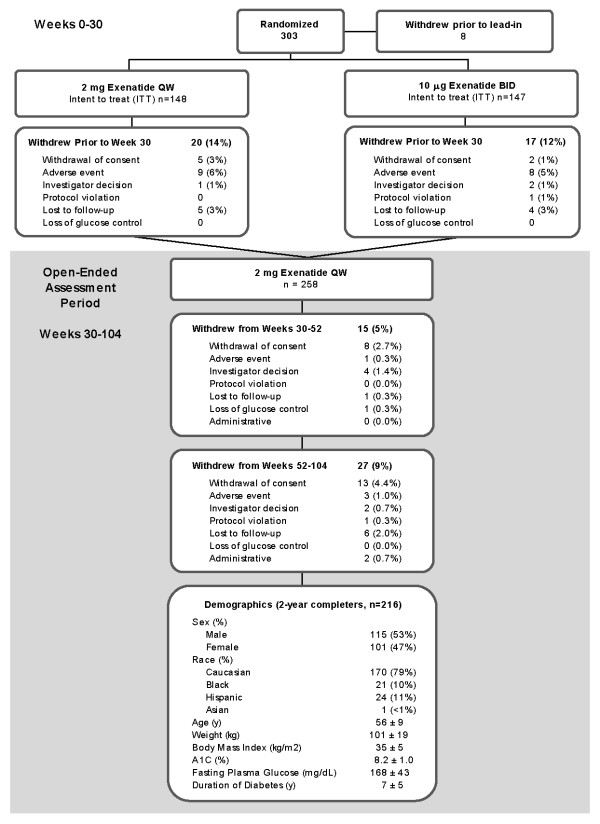
**Enrollment, patient disposition, and baseline characteristics**. Of the 303 patients originally randomized to the study, 295 comprised the ITT population and 258 entered the subsequent 74-week assessment period (ITT population). Forty-two patients withdrew during the 74-week open-ended assessment period, resulting in a completer population of n = 216. The disposition and demographic characteristics of the 30-week and 52-week populations have been published previously [25,26].

Demographic characteristics of the completer population (n = 216) are described in Figure 1. Of the 295 patients included in the ITT population, 73% completed 2 years of treatment (Figure 1). The most common reason for study withdrawal was withdrawal of consent. The demographic characteristics of the ITT population (sex, 53% male; mean age, 55 y; Caucasian, 78%; Black, 10%; Hispanic, 12%; Asian, <1%; mean weight, 102 kg; body mass index, 35 kg/m^2^; A1C, 8.3; FPG, 169 mg/dL; duration of diabetes, 7 y) were nearly identical to the 2-year completer population.

### Effects on glycemic control and body weight

**Figure 2:**
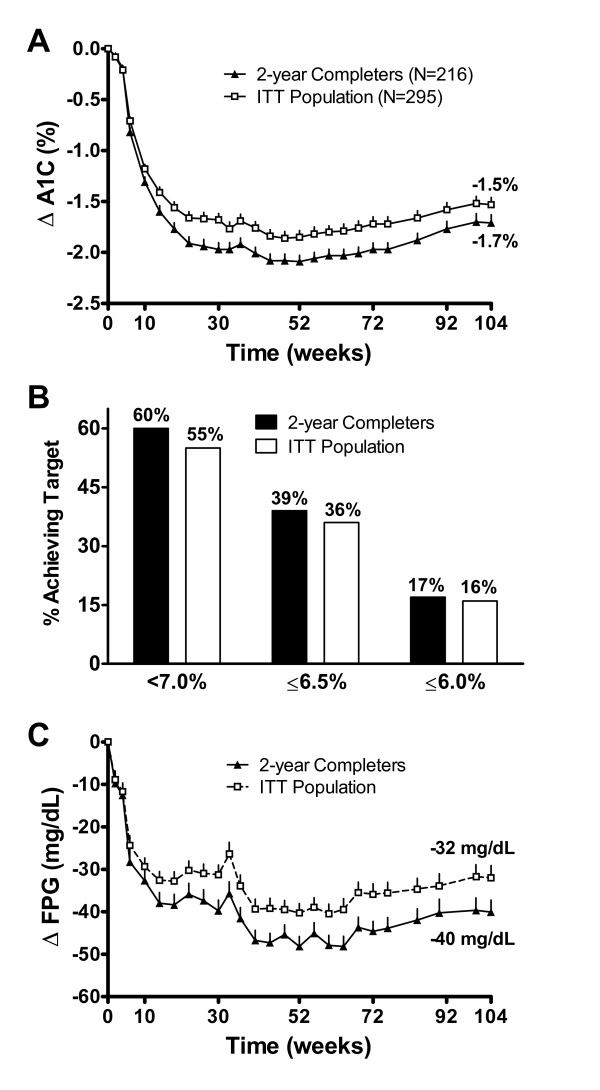
**Improvements in glycemic control over 104 weeks in the completer population (N = 216) and intent-to-treat population (N = 295)**. **A) **LS mean ± SE changes in A1C over 104 weeks. Baseline (BL) was 8.2% in the 2-year completer population and 8.3% in the ITT population. **B) **Proportion of patients who achieved A1C targets of <7.0%, ≤6.5%, and ≤6.0%. **C) **LS mean ± SE changes in fasting plasma glucose over 104 weeks. Baseline was 168 mg/dL in the 2-year completer population and 169 mg/dL in the ITT population.

**Figure 3:**
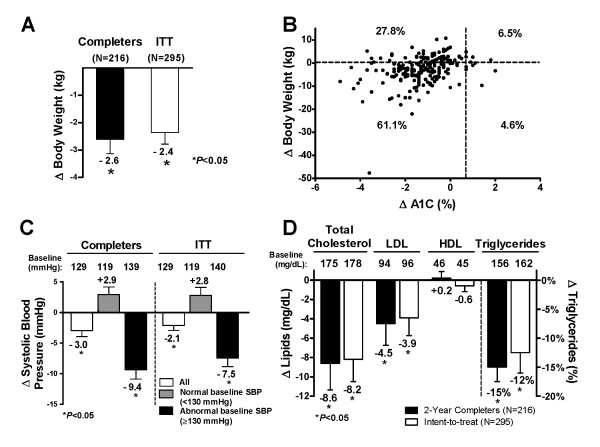
**Improvements in body weight, blood pressure, and serum lipids**. **A) **LS mean ± SE changes in body weight at 2 years for the ITT (N = 295) and completer populations (N = 216). **B) **Reductions in both body weight and A1C in the completer population after 104 weeks. **C) **LS mean change from baseline ± SE in systolic blood pressure (SBP) after 104 weeks of treatment, further analyzed by normal or abnormal baseline SBP, in the completer population (N = 216; Normal baseline, n = 113; Abnormal baseline, n = 103) and ITT population (All, N = 295; Normal baseline, n = 158; Abnormal baseline, n = 137). **D) **Changes in serum lipids were seen at week 104 (completer and IIT populations): data are presented as LS mean ± SE for all lipids except for triglycerides (geometric LS mean percent change ± SE from baseline).

The results reported here for the completer population and ITT population consist of all patients regardless of initial randomization. Overall, patients in the 2-year completer population maintained improvements in A1C (Figure 2A), with LS mean ± SE (95% CI) change from baseline A1C of -1.7 ± 0.08% (-1.86 to -1.55%). The mean change from baseline A1C in the ITT population was -1.5 ± 0.07%. At the end of the 2-year assessment period, 60% of patients achieved A1C of <7.0%, 39% achieved A1C of ≤6.5%, and 17% achieved normoglycemia (i.e., ≤6.0%) (Figure 2B). The percentage of patients in the ITT population who achieved these glycemic goals were similar to those in the completer population and are shown Figure 2B. Overall, 89% of patients in the completer population achieved a reduction in A1C at year 2 (Figure 3B). In patients who received 2 years of treatment, FPG decreased by 40.1 ± 2.9 mg/dL (-45.7 to -34.5 mg/dL) (Figure 2C).

Body weight reduction was generally maintained during the study; at the end of the 2-year assessment period, body weight (mean ± SE [95% CI]) was reduced by 2.6 ± 0.5 kg (-3.64 to -1.58 kg) in the completer population and 2.4 ± 0.4 kg (-3.19 to -1.53 kg) in the ITT population (Figure 3A). The distribution of the change in A1C with respect to changes in body weight is shown in Figure 3B for the completer population. At 2 years of treatment, 66% of patients experienced weight loss and 61% achieved reductions in both A1C and body weight.

### Effects on blood pressure and fasting lipids

Significant improvements in systolic blood pressure (SBP) were observed in patients treated with exenatide QW (Figure 3C): the mean change ± SE (95% CI) from baseline was -3.0 ± 1.0 mmHg (-4.9 to -1.1 mmHg) in the completer population and -2.1 ± 1.0 mmHg (-3.8 to -0.5 mmHg) in the ITT population. Improvement in diastolic blood pressure (data not shown) was also significant in the completer population (-1.5 ± 1.0 mmHg [-2.8 to -0.2 mmHg]), but not in the ITT population (-0.8 ± 0.6 mmHg [-2.0 to 0.3 mmHg]). Improvements (LS mean change [95% CI]) in serum lipid profiles were demonstrated after 2 years of treatment (completer population), with clinically significant reductions from baseline in total cholesterol (-8.6 ± 2.8 mg/dL [-14.0 to -3.1 mg/dL]), low-density lipoproteins (LDL; -4.5 ± 2.2 mg/dL [-8.9 to -0.01 mg/dL]), and triglycerides (geometric LS mean percent change ± SE -15 ± 2.7% [-21% to -10%]; Figure 3D). Similarly, changes in the ITT population were -8.2 ± 2.3 mg/dL (-12.7 to -3.7 mg/dL) for total cholesterol, -3.9 ± 1.8 mg/dL (-7.5 to -0.3 mg/dL) for LDL, and -12 ± 2.7% (-21% to -10%) for triglycerides.

### Safety and tolerability

Frequent (≥5%) TEAEs that occurred for the first time or worsened after receiving exenatide QW from weeks 0 to 30 and weeks 30 to 104 (ITT population) are described in Table 1. The adverse event (AE) profile for exenatide BID treatment from weeks 0 to 30 was previously reported [25]. Overall, the types of events reported with exenatide were similar for the controlled (weeks 0-30) and uncontrolled (weeks 30-104) portions of the trial, with no new tolerability or safety issues identified. Nausea was the most common AE throughout the trial and was predominantly mild in intensity; no severe nausea was reported. The proportion of patients who experienced nausea decreased during the second phase (weeks 30-104) of the trial: the incidence of nausea during the initial 30 weeks of treatment was 27% in the exenatide QW group and 35% in exenatide BID group, both of which decreased to 12%, regardless of initial treatment, from week 30 to week 104. The incidence of injection site pruritis and injection site erythema also declined from week 30 (18% and 7%, respectively) to week 104 (4% for both), and no injection-site-related AEs led to withdrawal during the uncontrolled period. There were no episodes of major hypoglycemia. Of the 17 patients who experienced a minor hypoglycemic event between weeks 30 and 104, 16 were using a concomitant SFU. From week 30 to week 104, only 4 patients withdrew because of an AE: nausea, cholestatic hepatitis, positive antibody test, diabetes mellitus, and breast cancer. The incidence of serious AEs was generally low (11% at week 104) and there was no consistent pattern of reported events. Additionally, no cases of pancreatitis were reported.

**Table 1:** Frequent (≥5%) Treatment-emergent Adverse Events with Exenatide Once Weekly During Weeks 0 to 30 and Weeks 30 to 104

Adverse Event	Weeks 0 to 30 (n = 148*) (% of patients)	Weeks 30 to 104 **(n = 258**^**†**^**)** (% of patients)
Nausea	27	12

Vomiting	11	9

Diarrhea	15	12

Constipation	11	5

Dyspepsia	7	3

Gastroesophageal reflux disease	7	4

Injection site pruritus	18	4

Injection site erythema	7	4

Urinary tract infection	10	9

Gastroenteritis viral	9	7

Nasopharyngitis	7	16

Upper respiratory tract infection	8	24

Sinusitis	5	12

Influenza	1	5

Headache	6	5

Fatigue	6	4

Back pain	5	7

Arthralgia	5	6

Pain in extremity	1	6

*Patients randomized to exenatide QW.

^**†**^Patients who continued exenatide QW or switched from exenatide BID to exenatide QW.

## Discussion

Guidelines for the treatment of type 2 diabetes are currently focused on the use of agents that provide effective lowering of A1C, have beneficial effects on extraglycemic factors associated with long-term complications, and have favorable safety and tolerability profiles [9,28]. Recently, GLP-1 receptor agonists have been recommended as a treatment option for patients in whom hypoglycemia or weight gain are major concerns [9,11]. In particular, weight loss in type 2 diabetes is associated with improved insulin action and enhanced glycemic control [29], and it may favorably affect other comorbidites associated with diabetes such as hypertension and dyslipidemia [30].

The improvements in glycemic control that were previously reported with 52 weeks of exenatide QW treatment [26] were sustained in the present study in patients continuing exenatide QW treatment for 2 years, regardless of the initial 30-week randomization of treatment (i.e., QW or BID). After 2 years of treatment, reductions in A1C remained durable in all patients (LS mean A1C change, -1.7%). In addition to improvements in glycemic control, improvements were seen in several cardiovascular risk factors including body weight, blood pressure, and fasting lipids. Further studies are warranted to determine whether these improvements are associated with enhanced outcomes in patients with type 2 diabetes.

### Exenatide once weekly vs. exenatide twice daily

Previously reported results from this trial demonstrated that improvements in glycemic control achieved with exenatide QW were greater than those achieved with exenatide BID, with similar or greater improvements in other outcomes including body weight, blood pressure, and serum lipid profiles [25,26]. After 30 weeks of treatment, patients who received exenatide QW had a greater mean A1C reduction (-1.9% vs. -1.5%) than did patients who received exenatide BID, and more exenatide QW-treated patients achieved target A1C levels of <7.0% (77% vs. 61%) [25]. Of note, patients who switched from exenatide BID to exenatide QW at week 30 exhibited further improvements in glycemic control such that at week 52, all patients, regardless of initial treatment, exhibited similar mean A1C reductions (-2.0%) [26]. In the present analysis, the observed mean A1C reduction of 1.7% after 2 years of treatment suggests that glycemic control is maintained with exenatide QW, a finding that was also observed after 2 years of treatment with exenatide BID [20].

### GLP-1 receptor agonists and dipeptidyl peptidase-4 (DDP-4) inhibitors

Other GLP-1-related therapies currently available for the treatment of type 2 diabetes are the once-daily GLP-1 receptor agonist, liraglutide, and the once-daily DPP-4 inhibitors sitagliptin and saxagliptin [31-33]. The GLP-1 receptor agonists (exenatide BID or QW and liraglutide) have been shown to be more efficacious at providing glycemic control and promoting weight loss than the DPP-4 inhibitors, although with more gastrointestinal side effects than with DPP-4 inhibitors [31,34,35]. A comparison study between exenatide BID and liraglutide showed that both therapies had significant effects on A1C, as determined by a noninferiority comparison [31]; however, reductions were significantly greater with liraglutide (mean A1C change, -1.1%) than with exenatide BID (-0.8%). Weight loss was similar between exenatide BID and liraglutide (approximately 3 kg). At this time, the results of a comparison study of exenatide QW and liraglutide are not yet available (ClinicalTrials.gov Identifier NCT01029886).

### Safety and tolerability

Treatment with exenatide QW was safe and well tolerated during the 2-year assessment period, with a retention rate of 73%. Adverse events (AEs) were generally mild to moderate. Few patients who received exenatide QW withdrew due to an AE (1.4% in the uncontrolled period) and there were no withdrawals from the study due to serious AEs. Mild nausea was common, but the frequency and severity of nausea decreased or abated over time. To date, no safety signals specific to exenatide QW have been identified in clinical trials.

### Study limitations

There were several potential limitations of this study. The absence of a comparator arm during the 1.5-year uncontrolled period limits the conclusions that can be drawn from the results, and the open-label trial design inherently creates the potential for bias and can affect patient expectations. The trial design also complicates interpretation of the modest increases in A1C, FPG, and body weight seen from week 52 to week 104. Furthermore, the population was heterogeneous for background therapy with other antidiabetic drugs; however, the earlier results at 30 and 52 weeks in this study suggest that the data are consistent across populations, regardless of background therapy.

Results from both the ITT and completer populations were included in this study, and both populations present certain limitations to the interpretation of the results. For the ITT analysis, using a LOCF method is a conservative approach that accounts for early study withdrawal and provides a prediction of treatment effect for any subject who ever received treatment. The completer analysis mitigates the potentially large variations in treatment exposure by representing the actual efficacy for the full term of drug exposure. The results of the ITT analysis should, thus, be weighed against the results of the completer analysis, as efficacy data collected early in the trial (e.g., from subjects who withdrew early) may not be representative of the true treatment effect, which is established with increased duration of treatment exposure. However, subject retention is often a challenge in long-term trials and the results of the completer analysis may be affected by the composition of the completer population, which often includes a high proportion of subjects who experienced favorable outcomes with treatment and underrepresents subjects who had issues with tolerability or lack of efficacy.

## Conclusions

The present analysis demonstrated durable efficacy with long-term exenatide QW therapy and showed that common AEs, especially nausea, subsided with time. The continuous release of exenatide in the once-weekly formulation produced continuous GLP-1 receptor activation leading to sustained control of both fasting and postprandial hyperglycemia, which resulted in enhanced improvements in A1C. Additionally, the convenience of the once-weekly injection of exenatide offers an attractive alternative to once- and twice-daily injections, and could improve treatment adherence in the clinical setting [36]. This combination of effects suggests that the use of exenatide QW may be a viable, long-term option for the treatment of patients with type 2 diabetes.

## Abbreviations

AE: adverse event; BID: twice daily; BL: baseline; FPG: fasting plasma glucose; GLP-1: glucagon-like peptide-1; ITT: intent-to-treat; LOCF: last observation carried forward; LS: least squares; **QW: once-weekly; **SBP: systolic blood pressure; SC: subcutaneous; SD: standard deviation; SE: standard error; SFU: sulfonylurea.

## Competing interests

This study was funded by Amylin Pharmaceuticals, Inc. and Eli Lilly and Company. Dr. Gurney, Ms. Han, Dr. Pencek, and Dr. Walsh are employees of Amylin Pharmaceuticals, Inc. Dr. Taylor was previously an employee of Amylin Pharmaceuticals, Inc. Dr. Trautmann is an employee of Eli Lilly and Company. Amylin Pharmaceuticals, Inc. has a global agreement with Eli Lilly and Company to collaborate on the development and commercialization of exenatide. All authors own company stock and have stock options.

## Authors' contributions

KT, JH, and RP participated in the design of the analysis. JH and RP performed the statistical analysis. All authors were involved in the interpretation of the analysis. All authors were involved in drafting the manuscript or revising it critically for important intellectual content. All authors approved the final manuscript.

## Pre-publication history

The pre-publication history for this paper can be accessed here:

http://www.biomedcentral.com/1472-6823/11/9/prepub
